# Supplementary material for: Flatfish intestinal microbiota depend on various host traits, and vary with sediment type and bottom trawling effort
Source: Sci Rep. 2026 Jan 6;16:632. doi: 10.1038/s41598-025-34195-w (PMC12774904; doi:10.1038/s41598-025-34195-w)
Supplement: Supplementary file 1 — Supplementary Information. [file 41598_2025_34195_MOESM1_ESM.pdf]

Supporting information

Flatfish intestinal microbiota depend on various host traits, and vary with sediment type and bottom trawling effort

Michelle Gwinner[1]*, Holger Haslob[2], Hermann Neumann[2], Sahar Khodami[4], Peter J. Schupp[1,3], Guido Bonthond[1,2]*

[1] Institute for Chemistry and Biology of the Marine Environment (ICBM), School of Mathematics and Science, Carl von Ossietzky Universität Oldenburg, Ammerländer Heerstraße 114-118, 26129 Oldenburg, Germany
[2] Thünen Institute of Sea Fisheries, Herwigstraße 31, 27572 Bremerhaven, Germany
[3] Helmholtz Institute for Functional Marine Biodiversity at the University of Oldenburg (HIFMB), Ammerländer Heerstraße 231, D-26129 Oldenburg
[4] Senckenberg am Meer Wilhelmshaven, German Centre for Marine Biodiversity Research, Südstrand 44, 26382 Wilhelmshaven, Germany

* Correspondence: michelle.gwinner@uol.de, guido.bonthond@uol.de

Table S1. Model selection tables for Effective number of OTUs and richness.

	condition	log(age)	log(weight)	log2(grain)	sex	species	SAR	species : condition	species : log(age)	species : log(weight)	species : log2(grain)	species : sex	species : SAR	Df	Log Likeliood	AICc	Delta AICc	AICc weight
ENO	**-**	**-**	**-**	**-**	**-**	**-**	**+**	**-**	**-**	**-**	**-**	**-**	**-**	**4**	**-712.5**	**1433.3**	**0.00**	**0.087**
	-	-	-	-	-	-	-	-	-	-	-	-	-	3	-714.0	1434.1	0.84	0.057
	-	-	-	+	-	-	+	-	-	-	-	-	-	5	-712.2	1434.7	1.45	0.042
	-	+	-	-	-	-	+	-	-	-	-	-	-	5	-712.3	1435.0	1.72	0.037
	-	-	+	-	-	-	+	-	-	-	-	-	-	5	-712.3	1435.0	1.72	0.037
	+	-	-	-	-	-	+	-	-	-	-	-	-	5	-712.4	1435.2	1.92	0.033
richness	**-**	**+**	**+**	**+**	**-**	**+**	**-**	**-**	**-**	**-**	**+**	**-**	**-**	**10**	**-1058.1**	**2137.6**	**0.00**	**0.020**
	+	+	+	+	-	+	-	+	-	-	+	-	-	13	-1054.7	2137.9	0.27	0.017
	-	+	+	+	-	+	+	-	-	-	+	-	-	11	-1057.1	2138.1	0.44	0.016
	+	-	-	+	+	+	-	+	-	-	+	+	-	14	-1053.6	2138.1	0.48	0.015
	-	-	-	+	-	-	-	-	-	-	-	-	-	4	-1064.9	2138.1	0.51	0.015
	-	-	-	+	+	+	-	-	-	-	+	+	-	11	-1057.2	2138.2	0.59	0.015
	+	-	-	+	-	+	-	+	-	-	+	-	-	11	-1057.3	2138.3	0.71	0.014
	+	+	-	+	-	+	-	+	-	-	+	-	-	12	-1056.2	2138.5	0.87	0.013
	-	-	-	+	+	-	-	-	-	-	-	-	-	5	-1064.1	2138.5	0.89	0.013
	+	+	+	+	-	+	+	+	-	-	+	-	-	14	-1053.9	2138.6	1.00	0.012
	-	-	-	+	+	+	-	-	-	-	-	+	-	9	-1059.7	2138.6	1.02	0.012
	-	-	-	+	-	+	-	-	-	-	+	-	-	8	-1060.9	2138.7	1.12	0.011
	+	+	-	+	+	+	-	+	-	-	+	+	-	15	-1052.8	2138.8	1.22	0.011
	-	-	-	+	-	-	+	-	-	-	-	-	-	5	-1064.3	2139.0	1.38	0.010
	-	-	-	+	-	+	-	-	-	-	-	-	-	6	-1063.3	2139.1	1.44	0.010
	-	+	-	+	-	+	-	-	-	-	+	-	-	9	-1060.0	2139.1	1.48	0.009
	-	+	-	+	+	+	-	-	-	-	+	+	-	12	-1056.6	2139.2	1.60	0.009
	+	+	+	+	+	+	-	+	-	-	+	+	-	16	-1051.7	2139.2	1.61	0.009
	-	-	-	+	+	+	+	-	-	-	+	+	-	12	-1056.6	2139.2	1.63	0.009
	-	-	-	+	+	-	+	-	-	-	-	-	-	6	-1063.4	2139.3	1.68	0.008
	+	-	-	+	-	+	+	+	-	-	+	-	-	12	-1056.7	2139.4	1.83	0.008
	-	-	-	+	+	+	+	-	-	-	-	+	-	10	-1059.0	2139.5	1.91	0.008
	+	-	-	+	+	+	+	+	-	-	+	+	-	15	-1053.1	2139.5	1.92	0.008
	+	+	-	+	-	+	+	+	-	-	+	-	-	13	-1055.5	2139.5	1.94	0.007

Abbreviations: Effective Numbers of OTUs (ENO), condition factor (condition), median grain size (grain), swept area ratio (SAR), Akaike information criterion corrected for small sample sizes (AICc), degrees of freedom (Df).
Note: Only models with a delta AICc < 2 are shown. Station identity was included as random intercept in all models included in the selection procedure.

Table S2. ANOVA table ENO and richness

Response	predictor	χ^2	Df	Pr(>χ^2)[1]	
ENO	SAR	0.3055	1	0.08046	.
Richness	species	7.3252	2	**0.02567**	*
	log(age)	5.0468	1	**0.02467**	*
	log(weight)	3.8905	1	**0.04855**	*
	log2(grain)	7.7733	1	**0.00530**	**
	species : log2(grain)	6.7423	2	**0.03435**	*

Note: linear mixed effect models with station identity as random intercept
[1] *Significance codes: p < 0.1 (.), p < 0.05 (*), p < 0.01 (**), p < 0.001 (***)*

Table S3. Post-hoc pairwise comparisons on OTU richness

	Contrast	df	t-value	Pr(>t)[1]	
species	*B. luteum – L. limanda*	168	-1.517	0.1311	
	B. luteum – P. platessa	167	-2.525	**0.0302**	*
	L. limanda – P. platessa	158	-2.605	**0.0302**	*
species : log2(grain)	*B. luteum*	96.2	2	0.7767	
	L. limanda	116.9	1	**0.0052**	**
	P. platessa	137.8	2	0.0662	.

Note: P values are adjusted with the Holm-method.
[1] Significance codes: p < 0.1 (.), p < 0.05 (*), p < 0.01 (**), p < 0.001 (***)

Table S4. PERMANOVA table

	Df	SumOfSqs	R2	F	Pr(>F)[1]	
species	2	0.9556	0.0198	1.6778	**0.0007**	***
sex	1	0.2175	0.0045	0.7637	0.9088	
condition	1	0.4444	0.0092	1.5605	**0.0152**	*
log(age)	1	0.5223	0.0108	1.8341	**0.0025**	**
log(weight)	1	0.3190	0.0066	1.1200	0.2378	
log2(grain)	1	1.0312	0.0213	3.6208	**0.0001**	***
SAR	1	0.9932	0.0206	3.4877	**0.0001**	***
species : sex	2	0.6123	0.0127	1.0758	0.2757	
species : condition	2	0.7467	0.0155	1.3109	**0.0358**	*
species : log(age)	2	0.6433	0.0133	1.1294	0.1740	
species : log(weight)	2	0.5334	0.0110	0.9366	0.6326	
species : log2(grain)	2	0.5645	0.0117	0.9910	0.4788	
species : SAR	2	0.5773	0.0119	1.0136	0.4229	
Residual	141	40.1547	0.8311			
Total	161	48.3158	1.0000			

Note: Permanova based on Bray-Curtis distances
[1] Significance codes: p < 0.1 (.), p < 0.05 (*), p < 0.01 (**), p < 0.001 (***)

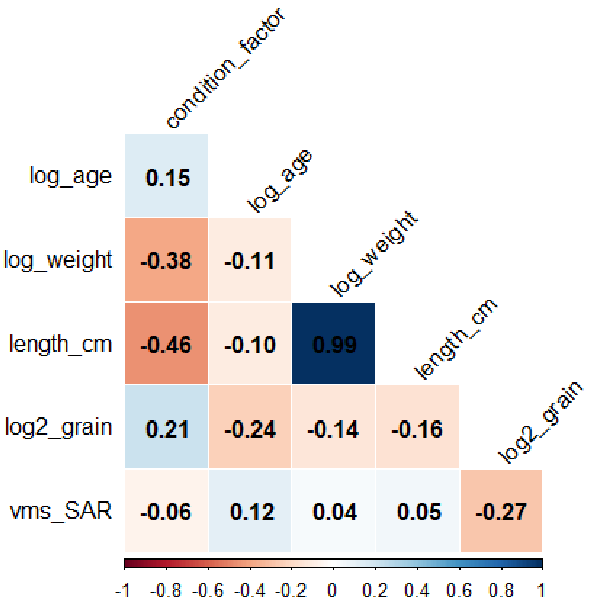

Figure S1. Raw spearman rank correlation values among all variables considered in the current study.

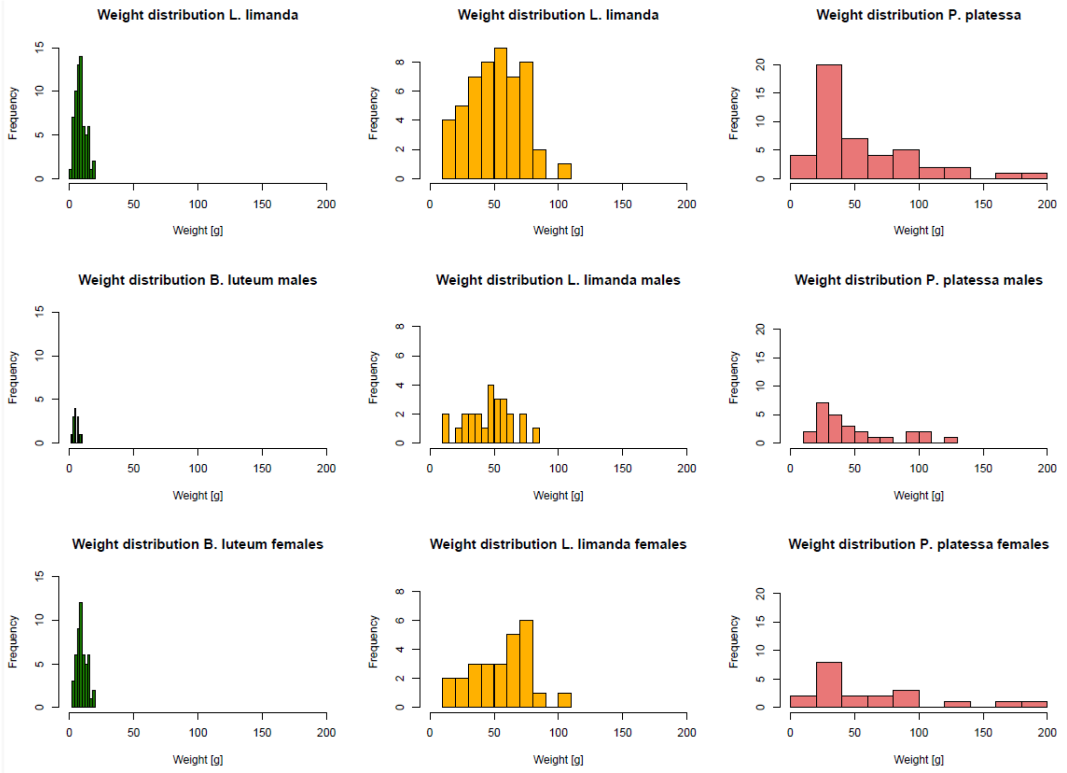

Figure S2. Histograms showing weight distribution ranges by species and sex

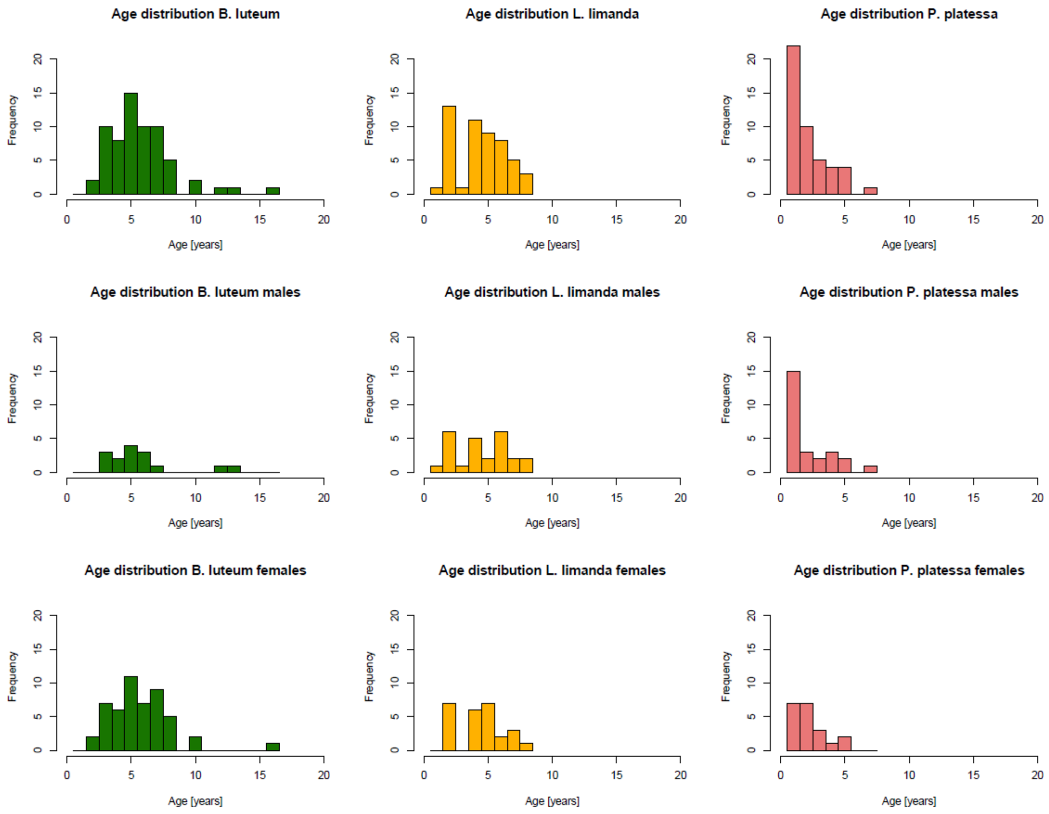

Figure S3. Histograms showing age distribution ranges by species and sex

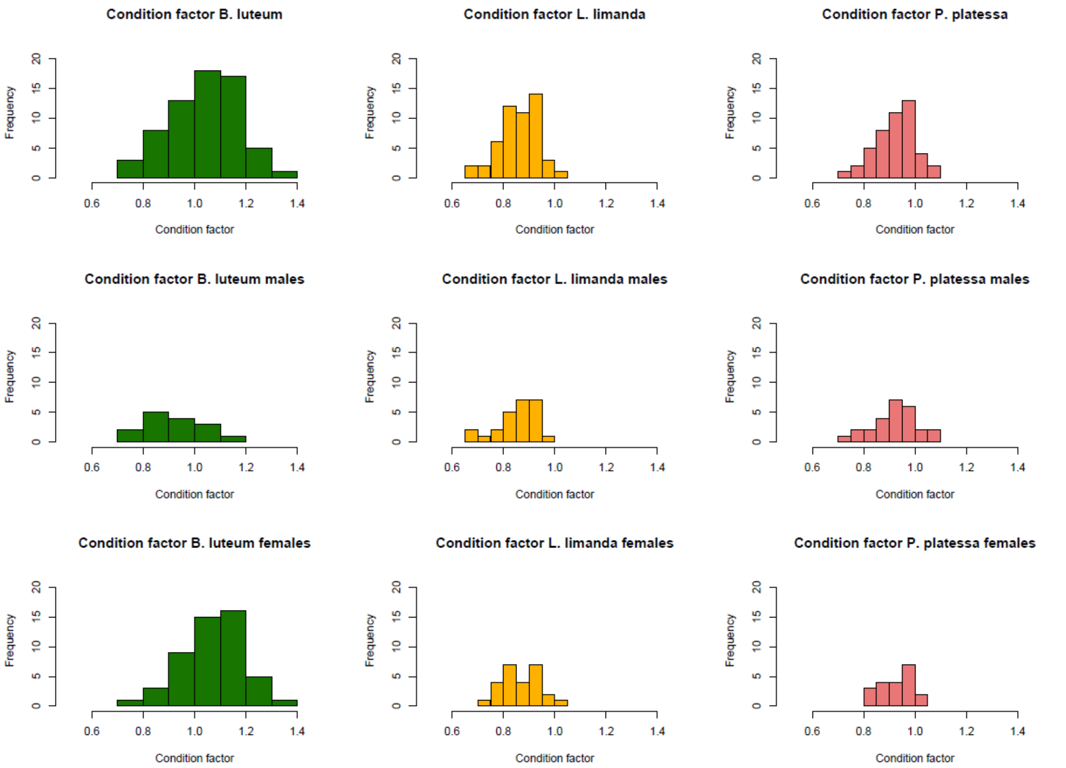

Figure S4. Histograms showing condition factor distribution ranges by species and sex

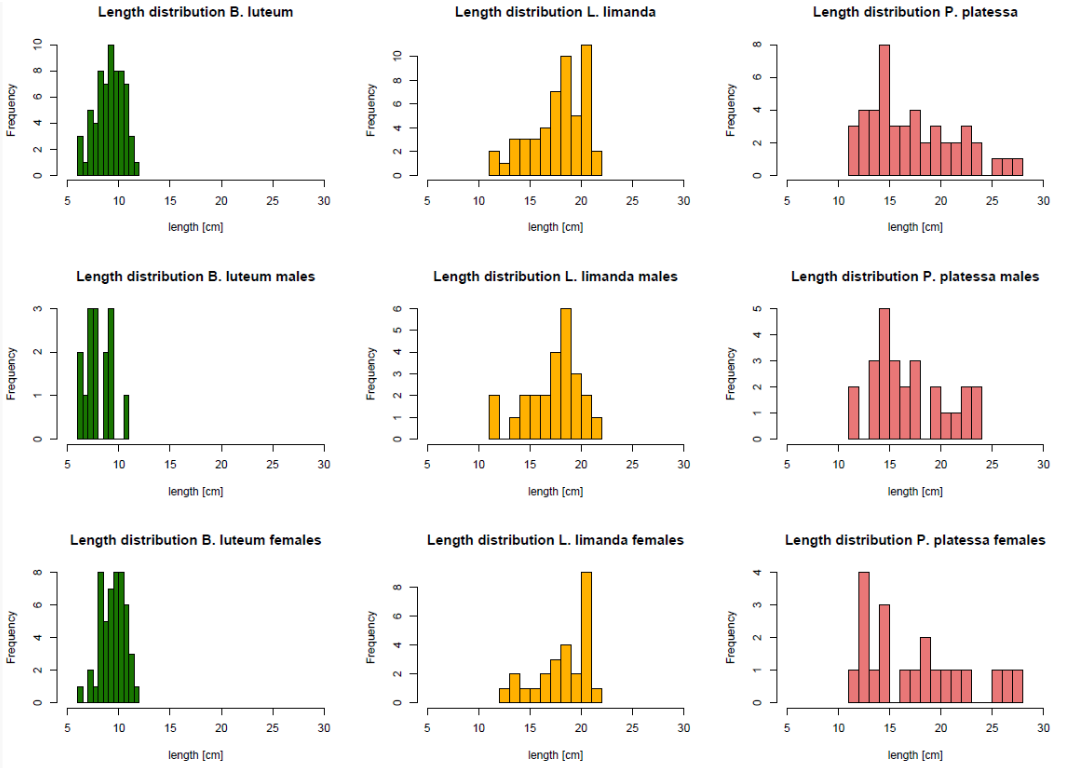

Figure S5. Histograms showing length distribution ranges by species and sex
